# ARE CAREGIVERS MORE LIKELY TO ENGAGE IN ADVANCE CARE PLANNING? EXAMINATION BY RACE AND ETHNICITY

**DOI:** 10.1093/geroni/igae098.0865

**Published:** 2024-12-31

**Authors:** Yifan Lou, Emily Mroz, Terri Fried, Emma Zang

**Affiliations:** Virginia Commonwealth University, New Haven, Virginia, United States; Emory University, Decatur, Georgia, United States; Yale University School of Medicine, New Haven, Connecticut, United States; Yale University, New Haven, Connecticut, United States

## Abstract

Spousal caregivers may become more aware of the value of advance care planning (ACP) as they witness care-recipients’ declining quality of lives. Due to unique experiences that often occur when caregiving for a spouse with dementia, dementia caregivers may be more motivated to engage in ACP. This presentation examines the association between spousal caregiving experience across dementia and non-dementia contexts and caregivers’ ACP behaviors and explores the moderating role of race/ethnicity. We identified 17,486 married individuals aged 65+ from the Health and Retirement Study 2000-2020. Three dimensions of ACP were considered: completion of a living will, designation of a Durable Power Attorney of Health Care (DPAHC), and having discussions with anyone about end-of-life care wishes. Logistic regressions compared ACP outcomes between 954 dementia caregivers, 3015 non-dementia caregivers, and 13,517 non-caregivers, controlling for sociodemographic and health covariates. Interactions between caregiver groups and race/ethnicity were examined. Spousal caregivers were significantly more likely to engage in all types of ACP compared to non-caregivers. Compared to non-dementia caregivers, dementia caregivers had significantly higher probabilities of completing a living will and designating a DPAHC. White caregivers reported highest probabilities of all three ACP outcomes, and being dementia caregivers further increased those rates. Positive association between caregiving experiences and ACP behavior were not observed in Black and Hispanic participants, except that being dementia caregivers increased the probability of having ACP discussions for Hispanic participants. We will discuss policy, practice, and research implications for the heterogeneous effects of caregiving experiences on ACP across racial/ethnic groups.

